# Synonymous mutations in oncogenesis and apoptosis versus survival unveiled by network modeling

**DOI:** 10.18632/oncotarget.8963

**Published:** 2016-04-25

**Authors:** Xiang Li, Yuan Chen, Hong Qi, Liyu Liu, Jianwei Shuai

**Affiliations:** ^1^ Department of Physics, Xiamen University, Xiamen 361005, China; ^2^ Complex Systems Research Center, Shanxi University, Taiyuan 030006, China; ^3^ College of Physics, Chongqing University, Chongqing 400044, China; ^4^ State Key laboratory of Cellular Stress Biology, Innovation Center for Cell Signaling Network, Xiamen University, Xiamen 361005, China

**Keywords:** TNF-α signaling network, parameter sensitivity analysis, mutation-induced oncogenesis, synonymous mutations, apoptosis versus survival

## Abstract

Synonymous mutations, which do not alter the encoded amino acid, have been routinely assumed to be ‘neutral’ and would have no effect on phenotype or fitness. Yet increasing observations have emerged to overturn this conventional concept. However, convicted elucidation of how synonymous mutations exert biological consequences in oncogenesis is still lacking. By performing systematic analysis of the TNF-α signaling network model, we identify the critical dose which separates the cell survival and apoptosis regions and define the sensitive parameters with single-parameter sensitivity analysis. Combining with the cancer-related mutation spectra obtained from 9 cancers, our results hint that, similar as missense and nonsense mutations, synonymous mutations are also strongly correlated with the parameter sensitivity of the critical dose, providing possible causal mechanism of the mutations in cancer development. Based on such a correlation, we furthermore dissect that members of caspases family proteases (caspase3, 6, 8) could jointly inhibit NFκB activation, providing efficient pro-apoptotic behavior. Thus, we argue that apoptosis module could suppress survival module through negative feedback of caspases family on NFκB.

## INTRODUCTION

As a conundrum, cancer is one of the most intensively studied biological phenomena. In 2011, Weinberg and Hanahan suggested there are 10 hallmarks for cancer [1]. Manifestation with these hallmarks, an important origin of cancerogenesis is somatic gene mutations [2]. Abundant genomic alterations in cancer genomes have been identified through the high-throughput whole-genome sequencing and massively parallel analyses [3, 4]. In cancer research, coding mutations that change the amino acid sequence are therefore the historically main efforts [5]. Synonymous mutations, which change the sequence of a gene without directly altering the amino acid composition of the encoded proteins, have been routinely assumed to be ‘neutral’ and would have no effect on phenotype or fitness, let alone induce oncogenesis over the past few decades. Nevertheless, this opinion has been in retreat [6–9].

Accumulating evidences began to suggest that synonymous mutations could apparently have some biological functional consequences. For example, Cartegni et al. [10] proposed that synonymous mutations could result in aberrant mRNA splicing. Kimchi et al. [11] demonstrated that synonymous coding Single Nucleotide Polymorphism (sSNPs) could affect protein conformation. In addition, sSNPs could also influence mRNA stability [12] and thus protein functions, which may lead to human diseases. A broad survey of disease-SNP associations indicated that non-synonymous SNPs and synonymous SNPs have a similar probability and a statistically equivalent effect size of disease association [13]. Acting as driver mutations [14], synonymous mutations may also contribute to human cancer [15, 16].

Despite the opinion has been overturned, the mechanism of synonymous mutations in cancer development is not unique [17]. Or more specifically, by now the experimental approaches to identify and validate these changes could not provide direct evidence for the mechanisms suggested. Sauna et al. [18] clearly pointed out that, considering the technical limitations, it is hard for current experiments to confidently demonstrate the mechanism by which even one synonymous mutation could cause cancer. Therefore, understanding the causal link between synonymous mutations and cancer initiation is a significant conundrum.

Apoptosis resistant with unexpected survival ability is one of the main hallmarks of cancer cells. The cell life-or-death decision is mainly governed by two modules, the survival and the apoptosis modules. Nuclear factor kappa B (NFκB), the core protein of the survival module, regulates cell survival through inducing the expression of numerous anti-apoptotic genes, such as FLICE-inhibitory protein (FLIP) and X chromosome-linked IAP (XIAP). FLIP, which contains a catalytically inactive caspase-like domain, interferes with the activation of caspase8 [19], an apoptotic initiator caspase; while XIAP inhibits caspase3, an apoptotic executioner caspase, through its second baculoviral IAP repeat (BIR) domain and NH_2_-linker [20]. Consequently, NFκB activation suppresses apoptosis in oncogenesis and tumour progression. Specifically, the over-expressed NFκB will function as a tumor promoter, while the loss-expressed NFκB will act as a tumor suppressor [21]. Furthermore, Nakanishi et al. [22] proposed the implication of NFκB inhibitors as sensitizers to anticancer drugs. Although the mutation-induced oncogenesis mechanism on how the survival module suppresses the apoptosis module has been largely studied, clinically more crucial issues are whether and how the apoptosis could compete or mediate the survival module, thus overcoming the anti-apoptotic effect and guaranteeing the apoptosis process to take place easily.

We therefore employ the approach of network modeling to investigate the possible mechanism of synonymous mutations in cancer development. In detail, we discuss the signaling pathway of tumor necrosis factor alpha (TNF-α), which can direct its signals down the survival and apoptosis modules. Ahead of this analysis, the signaling pathway should correctly catch the protein-protein interactions (PPIs). Nonetheless, though various high-throughput techniques have been applied to study PPIs in experiments [23-26], the reliability of the data is often concerned as several experimental limitations are presented. The PPIs are often activated under specific conditions in tissues while the experimental data are often gotten on certain condition or certain cell type [27, 28]. The large-scale screens treat proteins and genes as simple monolithic nodes in a pathway [29, 30] and the findings which were frequently based on the experiment with over-expression, loss-expression or dropout proteins [31] should be taken with caution as well. Moreover, false interaction data may also be suggested due to the limitations of analysis process in experiment [32, 33]. Hence, with possible missing and spurious PPIs, current data generated by these high-throughput experiments could partially reveal the biologically realistic picture of the cell.

Accordingly in this study, we aim to address the following two questions: (1) what mechanism could be for synonymous mutations in cancer development; (2) whether there are some possible additional PPIs in the signaling pathway, particularly between survival and apoptosis modules. By simulating the dynamic behavior of the TNF-α modulated signaling network, we determine the critical dose of TNF-α which separates the regions of cell survival and apoptosis. The critical dose reflects the threshold for cell apoptosis. The sensitivity of each model parameter can be determined by its modulation on the critical dose. Then, based on the cancer-related mutation database, we find a good correlation between the model parameter sensitivity and the corresponding mutated oncogenic genes. Such correlations indicate that, similar to missense and nonsense mutations, synonymous mutations could also change the dynamical parameters of the corresponding proteins in signaling network, and thereby increase the critical dose of TNF-α for cell death, ultimately facilitating oncogenesis and tumour progression. Furthermore, we also provide an approach to predict the possible feedback loops by integrating signaling network-based dynamic modeling with mutations spectra analysis. As a result, our comparison suggests that members of caspases family proteases (caspase3, 6, 8) could jointly provide negative feedback loops on NFκB, which is an efficient pro-apoptotic feedback mechanism of apoptosis versus survival modules.

## RESULTS

### Cell-fate governed by TNF-α signaling pathway

**Figure 1:**
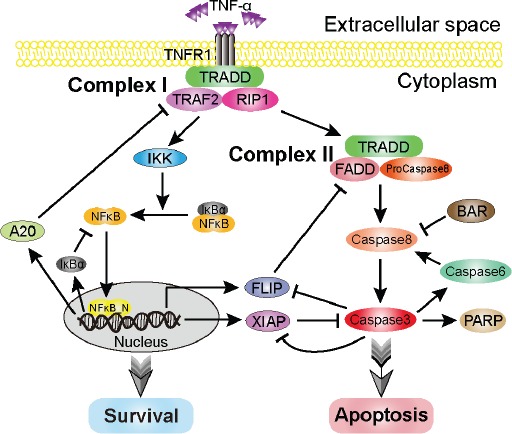
Schematic representation of the TNF-α-mediated cell-fate decision network Upon TNF-α stimulation, it binds with the receptor, TNFR1, and subsequently activates the survival and apoptosis modules. The core part of the survival module is NF-κB, which induces a variety of anti-apoptotic factors, such as FLIP and XIAP. The caspase8 and caspase3 are the major initiator and executioner in the apoptosis module. Arrows and bars indicate activation/transcription and inhibition, respectively.

**Figure 2:**
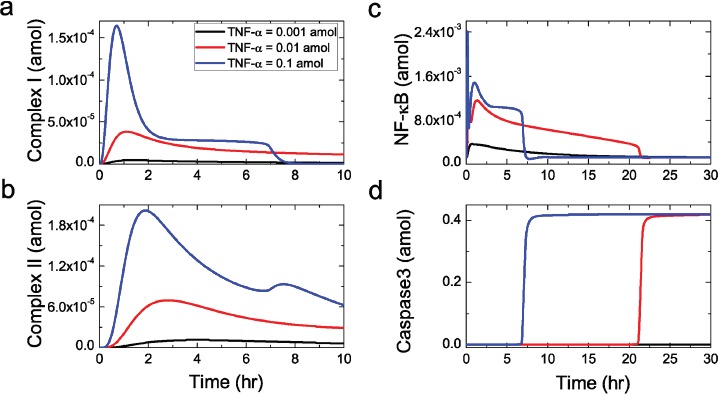
Sensitivity of the core protein activation to TNF-α stimulation The time evolution of Complex I **a.** Complex II **b.** NF-κB **c.** and Caspase3 **d.** respectively. Black, red and blue lines represent sustained stimulus with 0.001, 0.01 and 0.1 amol, respectively.

A schematic representation of the regulatory network model is depicted in Figure 1. To qualitatively reflect the dynamic behavior of the regulatory network, we first present an overview of the network model in response to three typical doses of sustained stimuli (10^−3^, 10^−2^ and 10^−1^amol) of TNF-α. The time courses of four key protein concentrations are plotted in Figure 2. Our simulation shows that both the formation of Complex I (Figure 2a) and Complex II (Figure 2b) exhibit a dose-dependent kinetics. For a low TNF-α dose (10^−3^ amol), the concentrations of Complexes I and II keep low and the regulatory network barely shows any response (black lines). While a high dose of 10^−2^ amol leads to a rapid formation of the complexes (red lines). With an increase of TNF-α dose, the maximum concentrations of the complexes show a continuous increase. As defined by the model, the formation of Complex I naturally occurs earlier than that of Complex II, which is in agreement with the experimental observations [34]. Besides, Figure 2b indicates that the formation of Complex II can be initialized only several minutes after the loading of TNF-α stimulation.

As shown in Figure 2c, TNF-α activates NFκB, the core protein of survival module, via a rapid, dose-dependent kinetics as well. Then, activated NFκB translocates into the nucleus to alter the anti-apoptotic genes expression, such as FLIP and XIAP. Such process has been proved in skeletal muscle [35]. Experimental studies have revealed that most cells survive at low dose of TNF-α, while high dose of TNF-α induces apoptosis [36]. Similar behavior can also be observed in the model, as shown in Figure 2d. Here, the concentration of caspase3 which is the main executioner in apoptosis module is considered to reflect whether apoptosis is triggered. At a low TNF-α dose (10^−3^ amol), the caspase3 concentration remains at low level (black line), suggesting that apoptosis has not been triggered. While a high TNF-α dose (10^−2^amol) leads to the activation of caspase3. The concentration of activated caspase3 increases to a high level rapidly after a delay of few hours (red line), indicating the occurrence of apoptotic process in the cell. Additionally, in Figure 2d, compared with the 10^−2^ amol stimulus, a 0.1 amol stimulus leads to an earlier activation of caspase3 (blue line), suggesting that the caspase3 cleavage is faster upon stronger stimulation. Such a result is also in agreement with the experimental observations by Rehm et al. [37].

### Parameter variation and the critical dose

Single-cell imaging studies have demonstrated that activation of execution-caspases is a rapid, all-or-none process while apoptosis occurs [38]. With the quantitative approach of network modeling, mathematical simulations have attested that both XIAP and cytochrome c released from mitochondria control this all-or-none response [39, 40]. Whether the stimulation of TNF-α controls the all-or-none response of casapse3 has not been conducted. To deal with such a dubiousness, the reliable range of TNF-α stimulation dose in the network should be determined first. In vitro experiments, the stimulation dose of TNF-α is typically in the range of 10-100 ng/mL (0.6-6 nmol/L) [34] and then the corresponding range is considered to be 0-10^−2^ amol for TNF-α in our model.

**Figure 3:**
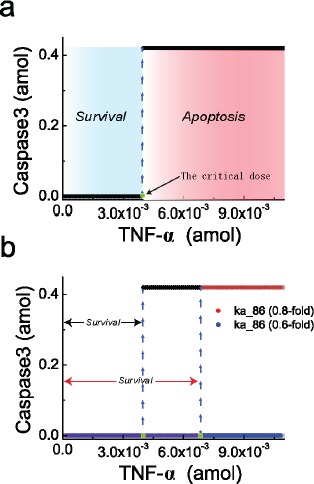
Steady-state behavior of caspase3 responding to TNF-α stimulation **a.** Steady-state curve of caspase3 for the signaling network responding to TNF-α shows a critical dose which can separate the regions of cell survival (blue region) and apoptosis (red region). **b.** Reducing the parameter ka_86 shifts the critical dose to the right, indicating an increasing apoptosis threshold. Black line represents the standard ka_86 without any change; red with 20% decrease; blue with 40% decrease. The blue dashed lines indicate the switch of steady-state of the activated caspase3.

Treating the TNF-α dose as the control parameter, the responding steady-state of activated caspase3 concentration is presented in Figure 3a. Similar to experimental observation, the modeling signaling network exhibits a rapid, all-or-none behavior for caspase3. Starting from the resting state, the activated caspase3 concentration remains low with low TNF-α dose. However, when TNF-α dose increases beyond a critical dose (about 0.004 amol), the activated caspase3 concentration switches to a high steady state, indicating the occurrence of apoptosis in the cell. As a result, the critical dose of TNF-α reflects the threshold of cell death. At the low dose region ( < 0.004 amol), the concentration of activated caspase3 remains low and the cell is in the survival state (green area in Figure 3a). However, at the high dose region ( ≥ 0.004 amol), the cell is driven to the apoptosis state with a high concentration of activated caspase3 (red area in Figure 3a).

Obviously, in regulatory network, the regions of TNF-α for cell survival and apoptosis are separated by the critical dose, and such a critical dose could be significantly affected by certain parameters variation [41]. As an example given in Figure 3b, compared with the standard model, the 20% decrease of the caspase8 production rate (ka_68) in the network shifts the critical dose to the right. As a result, the survival region is increased and a strong TNF-α stimulation is required for initiating apoptosis. If ka_68 is further decreased by 40%, high level of caspase3 can never be reached even at extremely high dose of TNF-α. Then, cell apoptosis will not occur in this case. This result is consistent with the Western blot experimental observations [42], which demonstrated that caspase3 is a major target of caspase8 and cells can escape from apoptosis with inhibited caspase8. The cell with such an inhibited caspase8 condition provides possible chance to avoid apoptosis, which may facilitate oncogenesis and tumour progression. Actually, caspase8 gene expression is frequently silenced and hypermethylation of regulatory sequences has been detected in multiple cancers [43–45]. Taken together, the changes of cell condition that is embodied by the changes of model parameters, such as the decreased caspase8 production rate, could consequently shift the critical dose, resulting in the resistance of cell death and possible incentive for oncogenesis [46, 47].

### Parameter sensitivity and cancer-related mutations

Analysis of parameter changes has been successfully employed for investigating the correlations between gene mutations and oncogenesis. For instance, Stites et al. [48] investigated the common oncogenic mutations in the Ras signaling network by evaluating the responses of the steady-state concentrations to parameter variations. Furthermore, Chen et al. [49] recently found a strong correlation between parameter sensitivity and oncogenic mutations in the p53-induced apoptosis signaling network. This study demonstrated that parameters that significantly affect the network bifurcation point correspond to genes with high-frequency oncogenic mutations.

In our regulatory network, certain parameter change, such as the decreased caspase8 production rate (ka_68) in Figure 3b, could significantly undermine the apoptotic function, which possibly facilitate oncogenesis. Therefore, we have an assumption that variations of the sensitive parameters which can significantly increase the critical dose render the abnormal apoptotic function of signaling network and likely cause cancer. Thus, as cancer is a genetic disease, the sensitive parameters should typically correspond to the mutated genes in cancers.

**Figure 4:**
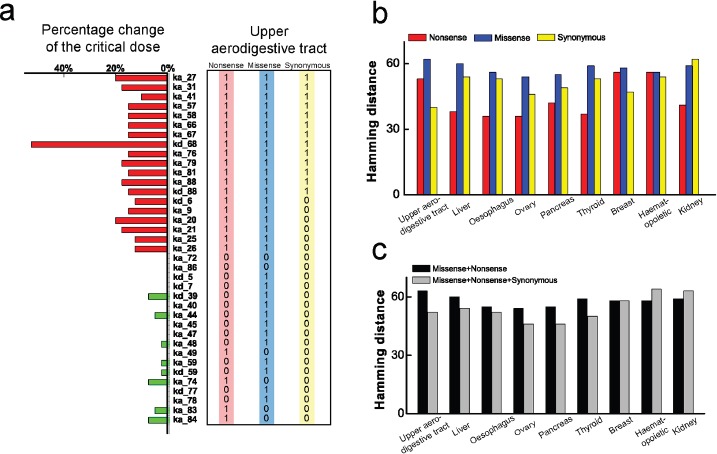
Comparison between parameter sensitivity and the corresponding cancer-related mutations **a.** Comparison between sensitivity spectrum of subset parameters and the corresponding genes spectra for three point mutations of upper aerodigestive tract cancer. Sensitive and insensitive parameters are marked by red and green stripes, respectively. The numbers in the frame are the Boolean variable which represents the genes with mutations (1) or without mutation (0). **b.** Calculated HDs between parameter sensitivity spectrum of all parameters and the genes spectra for three point mutations of 9 cancers. The red, blue and yellow bars represent the HDs corresponding to nonsense, missense and synonymous mutations, respectively. **c.** Calculated HDs between parameter sensitivity spectrum of all parameters and the genes spectra with combined action of the two and three mutations, respectively. The black bars correspond to the combined action of missense and nonsense; while the gray bars correspond to the three point mutations.

To explore the possible relationship between parameter sensitivity of the TNF-α signaling network model and the cancer-related mutations, we first select a subset of the parameters which are associated with the core proteins in the network, such as IKK degradation rate (ka_40), NFκB-IκBα complex degradation rate (ka_41), caspase3 activation rate (ka_79) and caspase8 activation rate (ka_80). Parameter sensitivity spectrum of the selected core proteins is plotted in Figure 4a, in which the sensitive and insensitive parameters are marked in red and green respectively. Secondly, we collect the cancer-related point mutations (missense, synonymous and nonsense mutations) from the upper aerodigestive tract cancer in COSMIC and convert to Boolean variables. We then compare the parameter sensitivity spectrum with the three point mutations in the corresponding genes, respectively. As shown in Figure 4a, almost all the sensitive parameters (marked by red stripes) are frequently corresponding to mutated oncogenic genes, but the insensitive parameters (marked by green stripes) are less corresponding to gene mutations. The insensitive parameters, which can hardly increase the critical dose of TNF-α to induce apoptosis, can hardly cause cancer and therefore should frequently correspond to genes without mutations. This result supports our assumption that parameters which render the network apoptotic dysfunction are strongly correlated with cancer-related mutations. And the result is basically consistent with the observation that the parameter sensitivity has an intimate relativity with oncogenic mutations [49].

As we known, somatic missense and nonsense mutations are well-recognized as the mainly cause of cancer, and the parameters change-induced apoptotic dysfunction in network could be arisen from this two point mutations. Recently, Zhao et al. [50] successfully dissected the causal mechanism of missense mutations-induced oncogenesis by conducting the parameter sensitivity analysis. Whereas, whether and what the causal mechanisms of synonymous mutations could induce oncogenesis is still an open issue. Here, in our study, through measuring the correlation between parameter sensitivity spectrum and the mutated genes, we can evaluate the association of the three point mutations, particularly for synonymous mutations, with cancer.

The achieved sensitivity spectrum of all parameters and the collected corresponding mutated gene spectrum are presented in the Supporting Information (Supplementary Table S1). As the somatic nonsense and missense mutations are definitely linked with oncogenesis, we first calculate the Hamming distance (HD) between the parameter sensitivity spectrum and the spectra of these two mutations, resulting in the value of 52 and 63, respectively. Subsequently, we calculate the HD between the parameter sensitivity spectrum and the spectrum of synonymous mutations, resulting in a surprisingly small value of 40. These results clearly indicate that, compared with nonsense and missense mutations, synonymous mutations indeed exhibit a better correlation with the parameter sensitivity of the signaling network.

Besides the upper aerodigestive tract cancer, more systematic analysis are conducted by collecting the three point mutations spectra from other 8 different cancers with high mutation rate, including liver, oesophagus, ovary, pancreas, thyroid, breast, haematopoietic and lymphoid tissue and kidney. Likewise, we calculate the HDs between the parameter sensitivity spectrum and the corresponding gene mutation spectra with the three point mutations for these 8 different cancers. As shown in Figure 4b, similar correlations with the parameter sensitivity spectrum are presented among the three point mutations. These results indicate that, similar as missense and nonsense mutations, synonymous mutations are also likely to be associated with parameter changes-induced network apoptotic dysfunction.

Strictly speaking, cancer is a consequence of combined action of several somatic mutation types. To further validate that synonymous mutations could be functional in apoptotic dysfunction, we then evaluate the correlation between the parameter sensitivity spectrum and the genes with combined action of mutations. Here, in the case of combined action of two mutation types, the gene is marked as “1” if at least one type mutation occurs; otherwise, marked as “0”. While for the combined action of three mutation types, the binarizing rule is that the gene is marked as “1” if at least two types show mutations.

We first consider the combined action of nonsense and missense mutations which have been corroborated in oncogenesis, and calculate the HDs between the parameter sensitivity spectrum and the corresponding mutated genes for the 9 different cancers. Then, we calculate the HDs in the case of the three point mutations combined action. As shown in Figure 4c, the HDs with two and three point mutations for the 9 different cancers are marked in black and gray, respectively. Remarkably, the comparison shows that, after considering synonymous mutations, the mutated genes show a better correlation with the parameter sensitivity spectrum particularly for the first 6 cancers, while the other 3 cancers show similar correlation. These results reveal that, besides the nonsense and missense mutations, the functional consequences of synonymous mutations in cancer development cannot be ignored.

Furthermore, for the combined action of three mutation types, we also discuss the case that the binarizing rule is that the gene is marked as “1” if at least one types show mutations. The comparison results are presented in Supplementary Figure S1. Even with such a binarizing rule, we can still draw the same conclusion that synonymous mutations could also correspond to the sensitive parameters in the regulatory network and disrupt the apoptotic function, ultimately facilitating oncogenesis and tumour progression.

### Caspases act as inhibitors of NFκB activation

**Figure 5:**
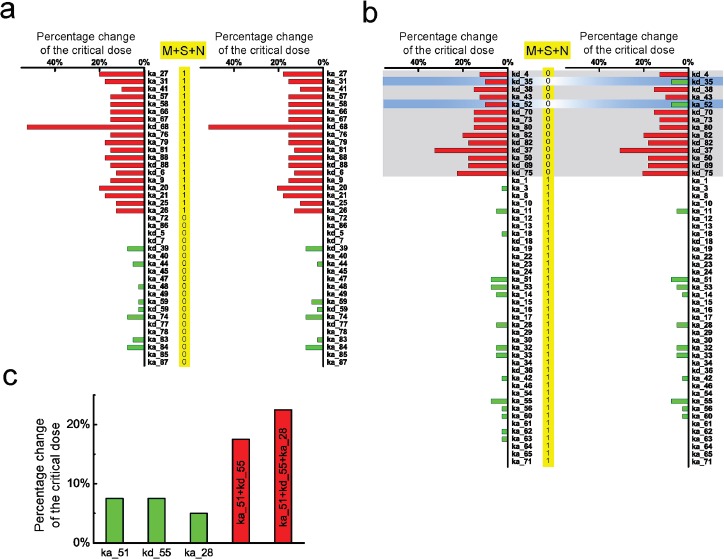
Comparison between parameter sensitivity of the modified model and the corresponding cancer-related gene mutation spectrum. **a.** The consistent subset of parameter sensitivity spectrum of the original model (left) and modified model with additional feedback (right). **b.** The inconsistent subset of parameter sensitivity spectrum of the original model (left) and modified model (right). The genes spectra are selected with the combined action of the three mutations. The gray region in (b) corresponds to 0 combined mutation, i.e. only 1 mutation out of three genes. Parameter sensitivity spectrum is not significantly influenced after considering the additional feedback except two parameters. In the gray region, two former inconsistent sensitive parameters (ka_52 and kd_35) become insensitive, resulting in consistent with the gene mutation spectrum. **c.** Comparison of parameter changes in isolation and co-occur. Three inconsistent insensitive parameters (marked in green), ka_51, kd_5 and ka_28, are changed at the two and three co-occur, respectively, resulting in a significantly accumulated influence on the change of critical dose (marked in red).

Based on above analysis, we propose that the mutated oncogenic genes, particularly for synonymous mutations show a good correlation with parameter sensitivity of signaling network. Nevertheless, there are some exceptions, resulting in a certain value of HD. As shown in Figure 5a and 5b, the parameter sensitivity spectrum of the network model and the corresponding gene spectrum with combined action of three point mutations are plotted. We separated the parameter sensitivity spectrum into two subsets, one for parameter sensitivity consistent with the mutated genes (Figure 5a) and the other one for inconsistent parameters (Figure 5b). Here for the combined action of three mutations, the binarizing rule is that the gene is marked as “1” if at least two types show mutations.

Such inconsistency might result from several factors. One factor is that a single gene mutation may not be enough to cause cancer. The multiple gene mutations could be essential in most cases [51]. Therefore, to discuss the cooperative effect of multiple gene mutations, we change several inconsistent insensitive parameters at the same time. As an example presented in Figure 5c, when varying the parameters of IKK activation (ka_51), Complex I inactivation (kd_55) and Complex II building (ka_28) individually, the critical dose is merely changed by 7.5%, 7.5% and 5%, respectively. However, if we vary the two insensitive parameters, ka_51 and kd_55 simultaneously, the critical dose is significantly changed by 17.5%. If we additionally consider the insensitive parameter, ka_28, the critical dose will be further changed by 22.5%. This result supports the viewpoint that cancers require the accumulation of a number of oncogenic mutations. Each mutation adds a certain advantage to increase cancer incidence, which is strongly correlated with the total number of divisions of the normal cells [52]. The co-occurring mutations could adequately explain the inconsistency of insensitive parameters. But, for the inconsistent sensitive parameters (the gray region in Figure 5b), such as the degradation rate of RIP1 (kd_4) and IKK (kd_35), variation of multiple parameters can hardly explain it. Therefore, some other factors may contribute to this inconsistency.

As current experimental data reveal only partial biologically realistic PPIs, we therefore speculate that another factor for the inconsistency may be due to the missing PPI in the signaling network model. To test our hypothesis, we discuss some possible interplay in the signaling network. Here we explore whether the apoptosis module could suppress or active the survival module. Consequently, we focus primarily on the interactions between NFκB and caspases which are the core proteins in the corresponding modules. We first propose a negative feedback loop of caspase8 on NFκB which may enhance apoptosis. Approach of adding the negative feedback loop is described in the section of Model and Methods. The biochemical parameter *k_negative_* is chosen as 0.6 *amol^−1^*s*^−1^* in this modified model. Parameter sensitivity of the modified model is presented in right columns of Figure 5a & 5b. Apparently, one can notice that, compared to the results with the original model in left columns of Figure 5a & 5b, the parameters sensitivity are scarcely influenced. The disparity is emerged in the gray region. Surprisingly, two parameters, the IKK degradation rate (kd_35) and IKK inactivation rate (ka_52), actually corresponding to non-mutation genes, which are defined as the sensitive parameters in the original model, become insensitive in the modified model (marked in green stripes in the right column of Figure 5b). Changes in Figure 5b indicate that, after adding the negative feedback, the model parameter sensitivity shows better consistency with the corresponding mutated genes. This result favors our hypothesis that a negative feedback loop of caspase8 on NFκB may be included in the signaling network.

**Figure 6:**
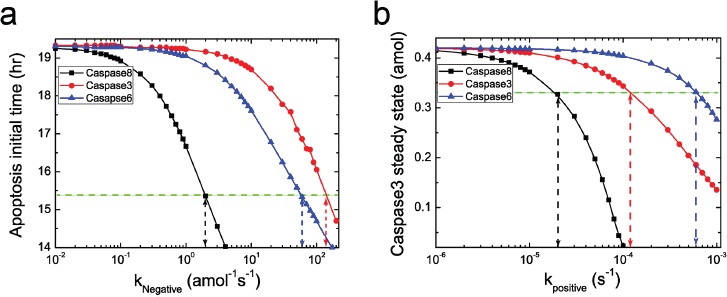
Effects of the two type feedback loops on apoptosis pattern **a.** Negative feedback loop of caspases on NFκB decreases the initial time of apoptosis. Black, red and blue lines represent the change of apoptosis initial time with the negative feedback strength of caspase8, 3 and 6 on NFκB, respectively. **b.** Positive feedback loop of caspases on NFκB decreases the steady state level of execution protein. Black, red and blue lines represent the change of steady state of caspase3 with the negative feedback strength of caspase8, 3 and 6 on NF-κB, respectively. The green dashed lines correspond to the 20% decrease of the corresponding pattern, which are used to determine the reasonable strength region for the caspases.

To systematically discuss the possible PPIs of caspases on NFκB, as shown in Figure 6, we first consider the negative and positive feedback loops of the three caspases, caspase8, 3, 6 with a quite broad range of feedback strength. Figure 6 indicates that additional negative feedback can decrease the initial time of cell death (Figure 6a). The greater the feedback strength is, the earlier the cell dies. Whereas the positive feedback loop decreases the steady state level of the executioner caspases (Figure 6b). The greater the positive feedback strength is, the lower the steady state level is. As a result, we distinguish the different dynamics caused by two interaction types, which are supported by the experimental observations that cells under different conditions mainly present two different respondence with the executioner caspases, i.e., the initial time of cell death and the level of steady state [53]. As shown in Figure 6 with green dashed lines, the reasonable ranges of feedback strength for caspases on NFκB are determined by limiting the affected range in 20%. As an example, for caspase8, the ranges are determined within 10^−3^~2 amol^−1^s^−1^ and 10^−6^~2×10^−5^ s^−1^ for negative and positive feedback, respectively. Our simulations show that, when considering the additional feedback loop within the determined range, location of the critical dose will be barely changed for both negative and positive interactions. The corresponding results are shown in Supplementary Figure S4.

**Figure 7:**
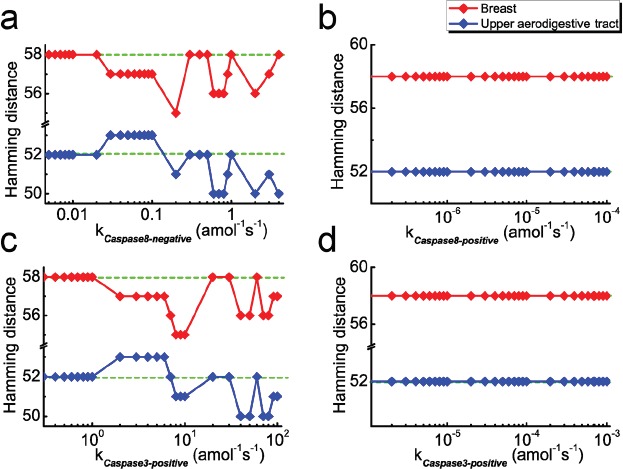
HD as a function of coupling strength after considering feedback loops **a.** The negative and **b.** positive feedback loops of caspase8 on NF-κB considered in the model, respectively. **c.** The negative and **d.** positive feedback loops of caspase3 on NF-κB considered in the model, respectively. Green dashed lines and red/blue rhombuses represent the HDs without and with considering the corresponding feedback loops, respectively.

Several reports have provided evidences that caspase8 plays important roles in NFκB activation upon various stimuli [54–56]. Nevertheless, during TNF-α-induced NFκB activation, the role of caspase8 is unclear yet. To answer this question, we consider the new models with additional feedback loop of caspase8 on NFκB, and subsequently calculate the HDs in the reasonable strength ranges. Since the cancer spectra are different, we first focus on the two cancers, upper aerodigestive tract and breast, as examples for discussion. The HDs with new models against feedback strength are plotted in Figure 7. Note that the green dashed lines represent the HD with the original model for the two cancers, resulting in the HDs of 58 and 52, respectively.

As shown in Figure 7a with the red line, the HD for new models are scarcely influenced with increasing negative feedback strength at the weak strength range (10^−3^ amol^−1^s^−1^< *k_caspase8-negative_* < 2×10^−2^ amol^−1^s^−1^) for breast cancer. However, at the relatively strong strength range (2×10^−2^ amol^−1^s^−1^ < *k_caspase8-negative_* < 4 amol^−1^s^−1^), the majority of the HDs are smaller than that of the original model. This tendency indicates that the parameter sensitivity of new model show better consistency with the mutated genes for breast cancer. In the case of upper aerodigestive tract cancer, a similar tendency could also been observed in the strength range of 2×10^−1^ amol^−1^s^−1^ < *k_caspase8-negative_* < 4 amol^−1^s^−1^, as shown in Figure 7a with the blue line. Note that our results show that these ranges are insensitive to the cut-off value.

Nevertheless, the tendency is not observed for the model with positive feedback. As shown in Figure 7b, compared with the original model, the HDs of new models with positive feedback are hardly influenced in the reasonable strength range for the two cancers. We therefore predict that, in both cancer cells, the initiator caspase, caspase8, could act as an inhibitor of NFκB activation in the signaling pathway through possible feedback loops.

Having discussed the case of caspase8, we are interested in the possible feedback of caspase3 on NFκB activation. In this case, the appropriate strength ranges are determined at 10^−1^~10^2^ amol^−1^s^−1^ and 10^−6^~10^−3^ s^−1^ for negative and positive feedback loops, respectively. Similarly, we calculate the HDs with the new models for the two cancers. Corresponding results are presented in Figure 7c and 7d, which are similar as the case of caspase8. As given in Figure 7c, HDs for new models with negative feedback are smaller than for the original model at the ranges of 2 amol^−1^s^−1^ < *k_caspase3_* < 10^2^ amol^−1^s^−1^ for breast cancer and 8 amol^−1^s^−1^ < *k_caspase3_* < 10^2^ amol^−1^s^−1^ for upper aerodigestive tract cancer, respectively. Whereas considering the new models with positive feedback, the HDs are barely influenced (Figure 7d).

Likewise, simulations are further carried out for caspase6, whose role in NFκB regulation has not yet been reported. Similar results of HD against coupling strength of feedback loops on NFκB are also obtained (see Supplementary Figure S2 in the Supporting Information). As a fact, the caspase3, caspase6 and caspase8 are tightly coupled to constitute a caspase module in the signaling network, and thus similar effects of feedback loops on NFκB should be naturally expected for these three caspases.

Besides the two cancers, we also calculate the corresponding HDs for the other 7 cancers (including liver, oesophagus, ovary, pancreas, thyroid, haematopoietic and lymphoid tissue and kidney) in Supplementary Figure S5 and Supplementary Figure S6. Apart from pancreas cancer, the results are consistent with above conclusion. In consequence, our discussion clearly shows that the caspases family proteases (caspase3, 6, 8) may jointly inhibit activation of the survival module core part, NFκB, through some PPIs to prevent the anti-apoptotic genes expression. This result further reveals that the apoptosis module is not merely restrained by the survival module as previously reported. In addition, it could also fight back to guarantee the strong and efficient pro-apoptotic activity in cells.

## DISCUSSION

Based on a systematic discussion of the TNF-α signaling network model, we first evaluate how cell fate responds to TNF-α stimulus and define a critical dose to describe the TNF-α threshold to cause cell apoptosis. By using a previously established method [49], we evaluate how the parameters modulate the critical dose of TNF-α and obtain the parameter sensitivity spectrum of the signaling network. Then, we observe a strong correlation between synonymous mutations and parameter sensitivity of the critical dose, providing an evidence of synonymous mutations in cancer development. Finally, through discussing such correlation, we propose a possible negative feedback of caspases on NFκB, suggesting a competition of apoptosis module with survival module in the signaling network.

### Synonymous mutations in cancer development

Over 50 human genetic diseases have been associated with synonymous mutations so far [18] and 5-10% of human genes are estimated to contain at least one harmful region because of synonymous mutations [57]. By employing the cancer-related mutations database, our study indicates that, compared to the two promised pathogenic mutations (i.e., nonsense and missense mutations), synonymous mutations show similar correlations with the parameter sensitivity of the signaling network, which renders the apoptotic dysfunction, providing the potential similar effect size of cancer association.

Our results are analogous to the previously statistical data [13] that non-synonymous SNPs (nsSNPs) and sSNPs shared similar likelihood and effect size for disease association by conducting a survey across 21429 associations between diseases and SNPs. The strong association observed in our study indicates synonymous mutations could cause cancer. The possible mechanism is that synonymous mutations could affect the speed and accuracy of genes translation, the stability of mRNA and so on. Such a modulation subsequently changes the biological functions of related proteins, corresponding to the parameter variations in the signaling network. Variations for sensitive parameters could cause a significant increase of the critical dose of TNF-α to enlarge the cells survival region, ultimately facilitating oncogenesis and tumour progression.

However, the network-based dynamic analyses ignore the structural details of proteins interactions and therefore we cannot clearly distinguish the causal molecular mechanism between the three mutations. A most recent study using molecular dynamics simulation has successfully provided the causal molecular mechanism underlying the correlation between parameter sensitivity of signaling network and missense mutations [50]. Thus, to prove the strong correlation between synonymous mutations and parameter sensitivity, the corresponding structural modulation of proteins at molecular basis should be further investigated in the future.

### Inhibition of caspases on NFκB

Previous studies have used genetic interaction correlation [58], phenotype similarity [59], phenotype correlation [60] and so on to predict PPIs. Actually, proteins often have intricate physicochemical dynamic connections, and interactions are condition-dependent. Thus, systematic approaches are needed and the interactions should be determined in a certain signaling pathway upon certain stimulus. Here, through coupling network dynamics with cancer mutations database, we propose that the apoptosis module could compete with the survival module. Specifically, we predict that the typical executioner caspases (caspase3, 6) and the typical initiator caspase (caspase8) could inhibit the survival module through negative feedback loops on NFκB, which may provide efficient pro-apoptotic activities in cells. Practically, growing experimental observations supported this predictions. Specifically, as a direct evidence, caspase3 could cleave the NFκB subunit p65/RelA [61–63], thus blocking the survival module activation and facilitating apoptosis. As an indirect evidence, caspase3 could cleave IkBa, generating a cleavage fragment to serve as a constitutive inhibitor of NFκB [64, 65]. Besides, caspase3 could also block NFκB activation by mediating activation of IKK [66].

Nevertheless, the role of caspase8 in mediating NFκB activation is controversial. A key role for caspase8 in TRAIL-, TCR-, dsRNA- and MDP-induced NFκB activation in various cell types has been claimed [67–70]. These observations implied that caspase8 could exhibit a positive feedback loop on NFκB. However, such opposite experimental results indicated that, during the stimulation of TNF-α, caspase8 cleaves RIP1, resulting in the abrogation of NFκB activation [71–73]. These experiments showed that caspase8 mediates NFκB activation in a stimulus-specific manner. In this paper, our study is based on the TNF-α signaling pathway, and the result is supported by the experiments which suggested caspase8 could indirectly suppress NFκB activation through cleaving RIP1. For caspase6, our results indicate that it may play a similar role as caspase3 and capase8 in inhibiting NFκB activation, which has not been reported with current experiments.

**Figure 8:**
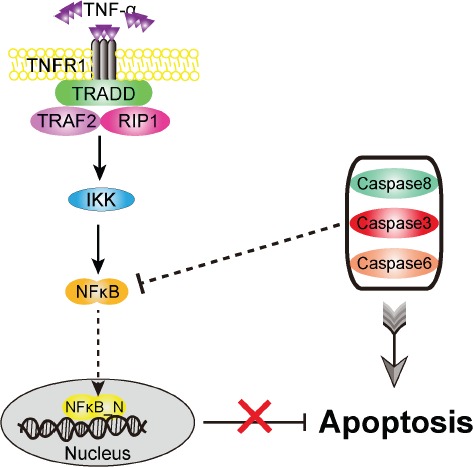
Proposed role of apoptosis versus survival modules in the signaling network Both the initiator and executioner caspases could jointly inhibit NF-κB activation upon TNF-α stimulus, providing a strong and efficient pro-apoptotic activity in cells.

Overall, as shown in Figure 8, we predict that, in the TNF-α signaling pathway, the initiator caspase (caspase8) and the executioner caspases (caspase3, 6) which are tightly coupled to be a caspase module may directly or indirectly inhibit NFκB activation to suppress the expression of anti-apoptotic genes. Such a suppression provides a potential mechanism to convert cells from survival to apoptosis. Thus, the cancer cells with inactivating caspases mutations might interdict not only the normal apoptosis process, but also the caspase-mediated NFκB inactivation. However, it is uncertain whether the caspase-mediated NFκB inactivation is the primary action of the apoptosis module on the survival module. Additional studies are needed to identify the other crucial proteins in the apoptosis module, such as FADD, TARDD and PARP, which might provide more possible potential targets for cancer therapy and prevention.

### Limitations of the network signaling modeling

There are several assumptions involved in our study. For example, one major assumption is that the model parameter sensitivity is related to gene mutations. By conducting bifurcation analysis, Chen et al. have studied the effect of parameter variation on dynamic properties and have successfully revealed the correlation between oncogenic mutations and parameter sensitivity in the apoptotic pathway that responds to DNA damage [49]. Such a conclusion serves as inspiration for us to assume that the fatal gene mutation should be typically related to sensitive parameters in the model.

Another major assumption is that the proposed positive and negative feedback loops could contribute to protein production and degradation. In the NFκB signaling network model [74] proposed by Hoffman et al., it has been suggested that the production and degradation reactions could be employed to reflect the positive and negative interactions, respectively. Besides, we also assume to choose ΔCd≥10%·Cd as the criterion in our analysis. For this assumption, we have drawn the ROC curves and calculated the corresponding Youden's indexes in Supplementary Figure S3, which suggests that the 10% cut-off rate could be employed.

As a fact, several limitations in this study need to be elaborated. Firstly, in the present study, we use a simplified network model to conduct our discussion. Such a network could not fully achieve the biologically realistic signaling pathway, as well as a precise predication. Therefore, we could just achieve a general, instead of a detailed conclusion. Nonetheless, the simplified model could still qualify the main features of the signaling pathway at the modular and molecular levels, such as the NFκB and caspase3 activation response to TNF-α stimulation (see Figure 2). To further validate our prediction, a comprehensive network model with the extended proteins, such as the cIAP, Nemo, Bcl-2, t-bid and so on, in the signaling pathway should be established in the further study.

Secondly, as cancer is a very complex genetic disease, numbers of cancer mutated genes have not yet been fully identified. Some genes which are currently thought to be without mutation could perhaps be identified with mutation in the future. Besides, whether the identified gene mutations are actually required for tumor development is not been determined. Driver- and passenger-mutations are the present point of view in cancer research [75]. Thus, the cancer-related mutations database does not provide sufficient information for our comparison by now. The prediction will be more convictive with a more precise database hereafter.

Finally, the corresponding rule of gene mutations with specific parameters is also simplified in our discussion. The full-information of each mutation on the network model parameters is not currently available. Mutation in one gene may impact multiple aspects of the role of the protein in the network, such as the protein functionality, binding capacity, or phosphorylation efficiency, and therefore should correspond to different parameters. The question is that it is still unclear whether one gene mutation could impact all, several or just one aspect of the protein. Therefore, owing to the limited information and the structural details of protein interactions, some important factors are not well-considered and the predication perhaps is not precise. However, the main goal of our study is to provide a possible causal mechanism of synonymous mutations in cancer development and to discuss the possible interplay in the signaling pathway. A more accurate comparison and convinced discussion including those factors should be conducted in the future work.

Nevertheless, in this paper, we predict the possible PPI by searching for a smaller HD between parameter sensitivity of signaling network and cancer-related mutation spectrum. We suggest that such searching approach can be applied to predict the missing interplay in other signaling networks for further experimental verification.

### Theories of cancer beyond gene mutation

The somatic mutation theory has been the prevailing paradigm in cancer research for the last 50 years [2]. It main premise claims that most cancers are caused by DNA mutations which alter the genes that regulate cell normal function. As a result, the great majority of modern cancer researches focus on studying various pathways that control the cancer cells to grow and spread and attempting to develop inhibitors to those pathways from the viewpoint of cancer mutations. Although there are many remarkable achievements by now, many facets of cancer still remain indecipherable. Therefore, cancer itself cannot been fully explained by mutations alone and many other theories have been proposed, including the Metabolic theory [76], the immunostimulation theory [77], the tissue organization field theory [78] and the speciation theory [79]. From the viewpoint of regulatory network dynamics, Ao et al. proposed that the normal and cancer cells can be thought of as two different states of the molecular network [80]. Overall, to provide more effective treatment, the viewpoint of cancer should be diversification rather than gene mutations alone.

## MATERIALS AND METHODS

### Network description and construction

The TNF-α signaling network, which governs cell fate, has been extensively studied over the past decades [34, 81, 82]. The balance between cell survival and apoptosis is mainly attributed to two modules, the survival module and the apoptosis module. In this study, we discuss a regulatory network model of the TNF-α signaling pathway proposed by Schliemann et al. [83], which comprises above two linked modules. A schematic representation of the regulatory network model is depicted in Figure 1. Upon TNF-α binding to its receptor, TNFR1 and other three proteins (TRADD/TRAF2/RIP1) are recruited to form Complex I, which is the pedestal for activation of the survival and the apoptosis modules [34, 84]. On one hand, Complex I is essential for activating IKK. In resting cells, NFκB is sequestered in cytoplasm by association with IκBα. Activated IKK stimulates the phosphorylation of IκBα, resulting in the release of NFκB. Subsequently, the released NFκB translocates into nucleus, binds to DNA and then induces the transcription of numerous anti-apoptotic genes, such as FLIP and XIAP. One the other hand, Complex I also converts to Complex II, which contains TRADD, FADD and pro-caspase8. Pro-caspase8 becomes activated in Complex II, eventually resulting in the activation of caspase3 in the apoptosis module. Several proteins, including BAR, caspase6 and PARP, have also been implicated in the regulation of apoptosis. In short, the core signal of cell survival is NFκB, which induces the expression of anti-apoptotic genes; while the cell apoptosis mainly depends on the activity of caspases, such as caspase8 (the initiator caspase) and caspase3 (the executioner caspase).

Approach of network modeling by using ordinary differential equations (ODEs) is well established and has been widely used to quantitatively understand the cellular regulatory behavior [48, 74, 85, 86]. Here, based on above TNF-α signaling pathway, the constructed network model consists of 47 components, 88 reactions and 106 kinetic parameters. The cell state is described by the component concentrations (*C_1_, *C*_2_, …, *C*_47_*), and the biochemical reaction rates are dependent on these concentrations and the kinetic parameters (*k_1_, *k*_2_, …, *k*_106_*) according to the law of mass action. The model is formulated as a set of coupled ODEs, describing the time evolution of concentrations of proteins and complexes in terms of the following general equation:

$$dC_{A}/\text{dt}=\text{S}n_{\text{AB}}\times J_{B}$$

where *dC_A_*/dt** is the concentration changing rate of component A with time. *J_B_* represents the rate of reaction B, and *v_AB_* denotes the element of stoichiometric matrix [87] that links the reaction rates of *J_B_* with component A. The 106 kinetic parameters have been determined by fitting the experimental data. Detailed description of the model can be obtained from the Biomodels database as MODEL1112210000. Differences between transient and sustained TNF-α stimuli were performed in both experiments and simulation [83]. Since this signaling pathway presents a faster response to sustained TNF-α stimulation, we mainly consider the sustained stimulus in our model.

### Parameter sensitivity and cancer-related mutations database

The model is given with a set of fixed parameters and the effects of parameter variations on the dynamic properties are not evenly distributed. According to the model dynamics, a critical dose (Cd) of TNF-α can be defined to separate the cell fate between survival and apoptosis. To determine the parameters which have a significantly impact on the critical dose, single-parameter sensitivity analysis is conducted by varying all parameters +/− 20% from its default value. The increase of the critical dose means a larger threshold for cell apoptosis, which is likely to become cancer cell. Thus we focus on the parameter change (increase or decrease) which can increase the critical dose and record the corresponding increase (ΔCd). If the increase of the critical dose is shifted by a large amount, such parameter is marked as the sensitive parameter. In detail, we assume that Δ*Cd* ≥10% ·*Cd* corresponds to sensitive parameters, while Δ*Cd* <10% ·*Cd* corresponds to insensitive parameters. The reason for choosing 10% is given in Supplementary Figure S3.

It has been suggested that there is a correspondence between gene mutations and specific parameters changes in the signaling network model [49]. In the case of association, dissociation, degradation and enzymatic processes, the relevant parameter changes are likely related to corresponding gene mutation. In other words, if any one of the protein genes mutates, the relevant parameter in the biochemical reactions will be modulated. We exclude the parameters related to the protein production process, which are likely related to the gene amplification and deletion. As a result, there may be a relationship between the cancer-related mutations and the parameter sensitivity for the critical dose of TNF-α. To dissect this possible relationship, the three point mutations of 9 cancers (including upper aerodigestive tract, breast, liver, oesophagus, ovary, pancreas, thyroid, haematopoietic and lymphoid tissue and kidney) are chosen: missense, synonymous and nonsense mutations. The spectra of cancer-related mutations are collected from Catalogue of Somatic Mutations in Cancers (COSMIC) [88] which is a major resource of genetic variants in different cancer types.

### Boolean variable and hamming distance

To discuss the possible relationship between parameter sensitivity and cancer-related mutations, one has to consider the following limitations. Although an increasing number of mutated oncogenic genes have been identified and are being updated in the database, current data in the COSMIC are still not precise. Furthermore, as stated in Ref. [49], the parameters do not have one-to-one correspondence with gene mutations and the “mutation hot spot” cannot be quantitatively defined. Thus, for a robust comparison, we employ Boolean variable to reflect the number of mutated gene samples. In detail, we use “0” (false) to denote no mutation for the corresponding parameter, and “1” (true) to denote a mutated sample for the corresponding parameter. Similarly, Boolean variable is also carried out to reflect the parameter sensitivity: with “0” to denote insensitive parameters, and “1” to denote sensitive parameters. With such Boolean variables, the relationship between parameter sensitivity and cancer-related mutations can be simply measured by Hamming distance (HD), which is defined as the difference between two binary strings.

### Construction of the missing PPIs

If there is an interaction between two proteins in the network, the corresponding biochemical reaction should be proposed and the corresponding ODEs of the network model should be considered. In this study, different types of interactions between caspases and NFκB are considered, including positive and negative feedback loops. Here, we assume that positive feedback loop contributes to the protein production. For instance, if a positive feedback loop of caspase3 on NFκB is considered, biochemical reaction (1) should be added:

$$\varphi^{\text{caspase}3}\to \text{NFkB};$$ (1)

Based on the law of mass, the ODE to describe the change of NFκB concentration should be given as:

$$\frac{d\left[\text{NFkB}\right]}{\text{dt}}=\ldots \ldots +k_{\text{positive}}*\left[\text{caspase}3\right];$$ (2)

Here, [X] represents the concentration of protein X, ······ represents the terms interacting with other proteins, *k_positive_* is the biochemical parameter, k_positive_*[*caspase3*] is the changing term related to the positive feedback loop of caspase3 on NFκB.

Similarly, we assume that negative feedback loop contributes to the protein degradation. If a negative feedback loop of caspase3 on NFκB is considered, biochemical reactions (3) should be added:

$$\text{NFk}B^{\text{caspase}3}\to \varphi ;$$ (3)

Then the ODE should be given as:

$$\frac{d\left[\text{NFkB}\right]}{\text{dt}}=\ldots \ldots -k_{\text{negative}}*\left[\text{caspase}3\right]*\left[\text{NFkB}\right];$$ (4)

Here, *k_negative_*is the biochemical parameter and k_negative_*[*caspase3*]*[*NFκB*] is the changing term related to the negative feedback loop of caspase3 on NFκB.

### Concerns to predict missing PPIs

Prior to discussing the possible missing PPIs, some concerns need to be clarified: First, how to predict a possible PPI based on the comparison of correlation between parameter sensitivity and mutated genes. For a robust comparison, we use Hamming distance (HD) to define the correlation of Boolean variables. Therefore, after considering an additional PPI in the network, if HD becomes decreasing, it means that the parameter sensitivity shows better consistency with the mutated genes. In this case, we predict such an interaction may possibly be favored. However, if HD increases or is not influenced, we propose such an interaction may not exist.

The second concern relates to how to characterize the impact of different interaction types on the signaling network dynamics. Previous experimental study [53] revealed that cells under different conditions mainly present two different respondence with the executioner caspases, i.e., the initial time of cell death and the level of steady state. Here, in our analysis, by checking the change of the initial time of cell death and the steady state level of the system, we characterize the impact of positive and negative feedback loops of caspases on network dynamics.

The last concern is to determine the reasonable range of feedback strength. In principle, one should notice that the dynamic properties of the network should not be largely affected after considering the additional feedback. Accordingly, we determine the strength range by limiting the affected range in 20%.

## SUPPLEMENTARY FIGURES AND TABLES




