# Supplementary material for: Synonymous mutations in oncogenesis and apoptosis versus survival unveiled by network modeling
Source: Oncotarget. 2016 Apr 25;7(23):34599–616. doi: 10.18632/oncotarget.8963 (PMC5085179; doi:10.18632/oncotarget.8963)
Supplement: Supplementary file 1 [file oncotarget-07-34599-s001.pdf]

SUPPLEMENTAL MATERIAL

The corresponding rules for mapping mutation spectra and model parameters

Our study is based on a TNF-α signaling network model proposed by Schliemann et al. [1] and the point mutation database obtained from COSMIC [2]. There are 7 main biochemical reaction types in the present model, i.e., association, disassociation, activation, translocation, transcription, production and degradation. According to the method established by Chen et al. [3], the corresponding rules are mapped as following:

(1) In the case of association and disassociation, such as the binding reaction $A + B \xleftrightarrow{k_1/k_{-1}} AB$, either or both of the two genes, A and B, may affect the reaction process. Thus, we assume that the change of the association and disassociation parameters k_1 and k_{-1} in the model are likely related to gene mutations of both A and B proteins.

(2) For the biochemical reaction of activation, such as $C \xrightarrow{k_3} C^*$, the process is related with gene mutation of C protein. Thus, the change of model parameter k_3 is likely related to C gene mutation. If the activation of C is modulated by another protein D, such as $C \xrightarrow{D} C^*$, the relevant parameter change is related to C and D gene mutations.

(3) For the translocation reaction process, such as $E\_c \xrightarrow{k_4} E\_n$, which means the translocation of protein E from cytoplasm to nucleus, the change of relevant parameter k_4 is related to E gene mutation. Besides, for the transcription process, such as $\phi \xrightarrow{G} F\_mRNA$, the relevant parameter change is related to the gene mutation of modulator G protein.

(4) The degradation process of protein H is related to gene mutation of H protein. If such a process is modulated by another protein I, the change of relevant degradation parameter is related to gene mutations of H and I proteins. However, the protein production process is likely related to the gene amplification and deletion, which is irrelevant to gene point mutations. Therefore, we exclude the corresponding parameters in our study.

Hamming distance

Hamming distance (HD) between two binary strings with equal length is the number of positions in which the corresponding binary numbers are different. In our analysis, we use HD to define the relationship between parameter sensitivity spectrum and gene mutation spectrum. For example, supposing the mutation spectrum is "1$\underline{0}$1$\underline{1}$0$\underline{0}$" and the corresponding parameter sensitive spectrum is "1$\underline{1}$1$\underline{0}$01". As marked by the underline, those three bits are different between the two spectra, giving the HD of 3.

REFERENCES

1. Schliemann M, Bullinger E, Borchers S, Allgöwer F, Findeisen R, Scheurich P. Heterogeneity reduces sensitivity of cell death for TNF-stimuli. BMC Syst Biol. 2011; 5:204.
2. Forbes SA, Bindal N, Bamford S, Cole C, Kok CY, Beare D, Jia M, Shepherd R, Leung K, Menzies A, Teague JW, Campbell PJ, Stratton MR, et al. COSMIC: mining complete cancer genomes in the Catalogue of Somatic Mutations in Cancer. Nucleic Acids Res. 2011; 39:D945-D950.
3. Chen J, Yue H, Ouyang Q. Correlation between oncogenic mutations and parameter sensitivity of the apoptosis pathway model. PLoS Comput Biol. 2014; 10:e1003451.

SUPPLEMENTARY FIGURES AND TABLE

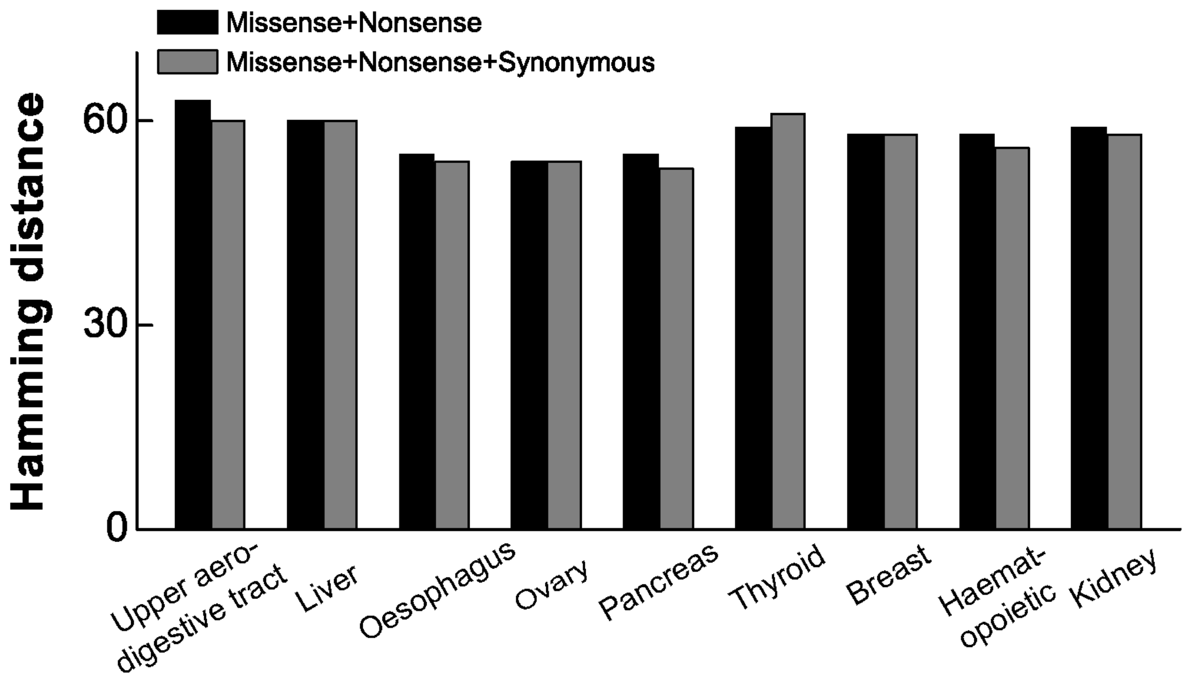

Supplementary Figure S1: HDs between parameter sensitivity spectrum of all parameters and the genes spectra. The black bars correspond to the combined action of two point mutations of missense and nonsense; while the gray bars correspond to the combined action of three point mutations. For both the combined actions of two and three mutations, the binarizing rule is that the gene is marked as “1” if only one type mutation occurs; otherwise, marked as “0”.

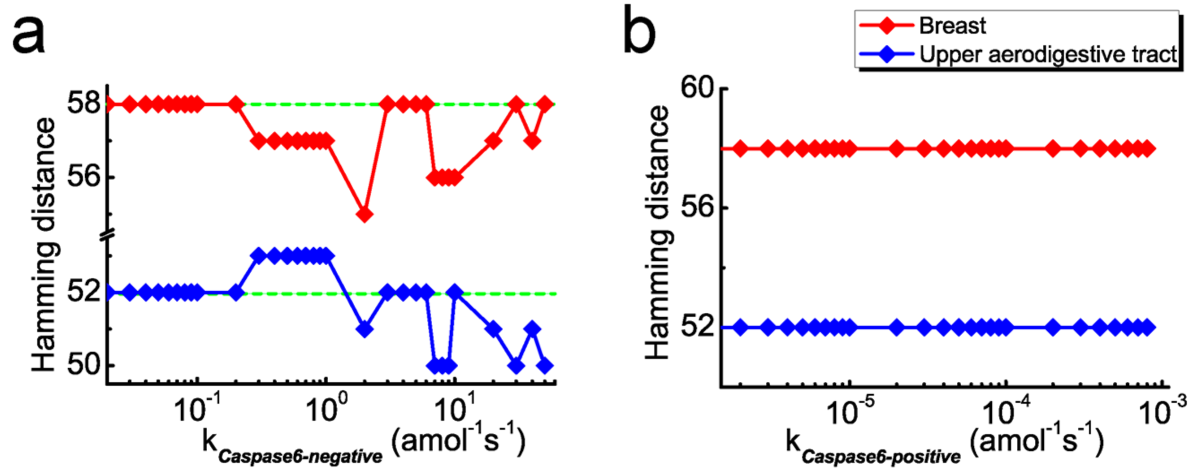

Supplementary Figure S2: HD as a function of coupling strength after considering feedback loops. a. The negative and **b.** positive feedback loops of Caspase6 on NFκB considered in the model, respectively. Green dashed lines and red/blue rhombuses represent the HDs without and with considering the corresponding feedback loops, respectively.

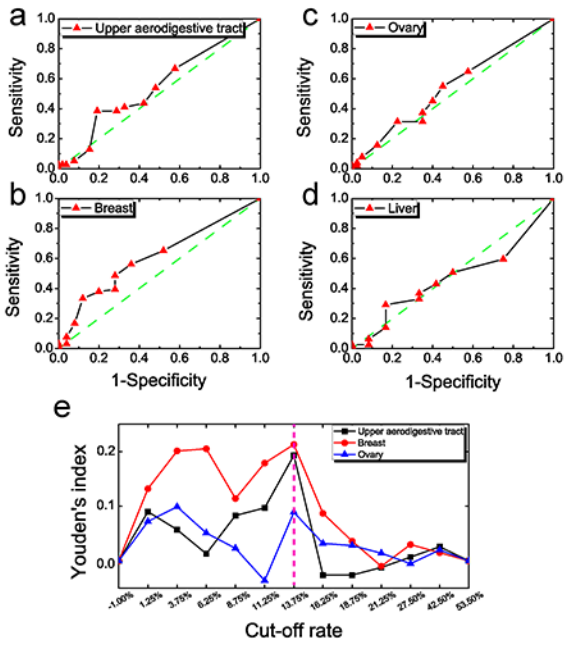

Supplementary Figure S3: The receiver operating characteristic (ROC) curve of cancers. The ROCs for **a.** upper aerodigestive tract, **b.** breast, **c.** ovary and **d.** liver cancers. **e.** The Youden's index against cut-of rate for upper aerodigestive tract, breast, and ovary cancers. For the three cancers, i.e., upper aerodigestive tract, breast and ovary, the maximal Youden's indexes are around 13.75%. For the rest of the cancers, such as liver shown in (d), it is hard to obtain nice ROC curves. Thus, for a simple comparison, we suggest a cut-off rate of 10% applied in our discussion.

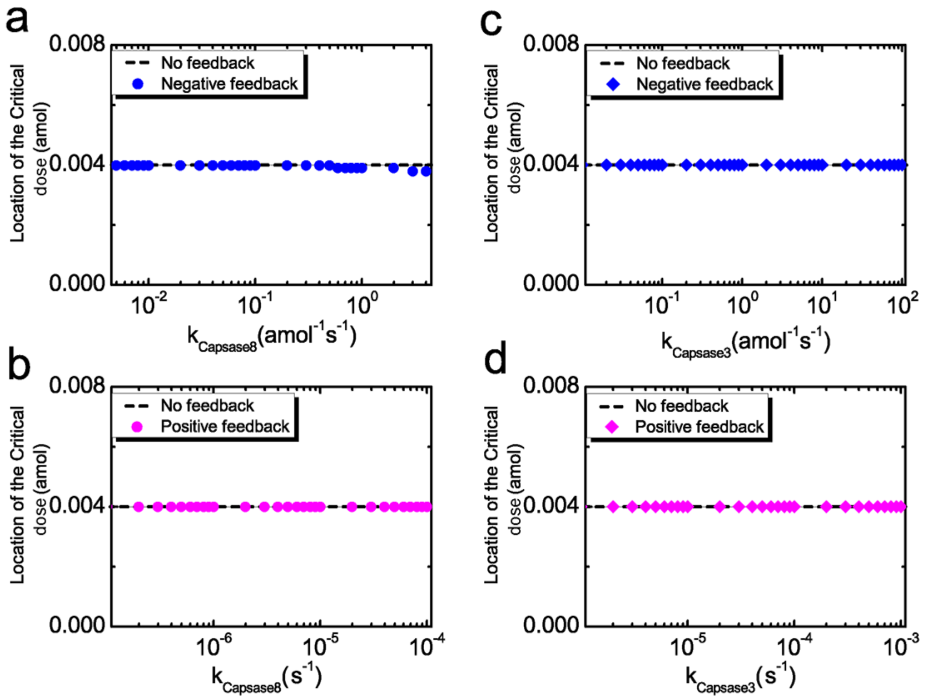

Supplementary Figure S4: Location change of the critical dose after considering the feedback loops. The location change of the critical dose against feedback strength after considering the negative **a.** and positive **b.** feedback loops of Caspase8 on NFκB, and after considering the negative **c.** and positive **d.** feedback loops of Caspase6 on NFκB. As a comparison, the dashed lines is for the value of critical dose without any feedback. The simulation results show that the added feedback loops could barely change the value of critical dose.

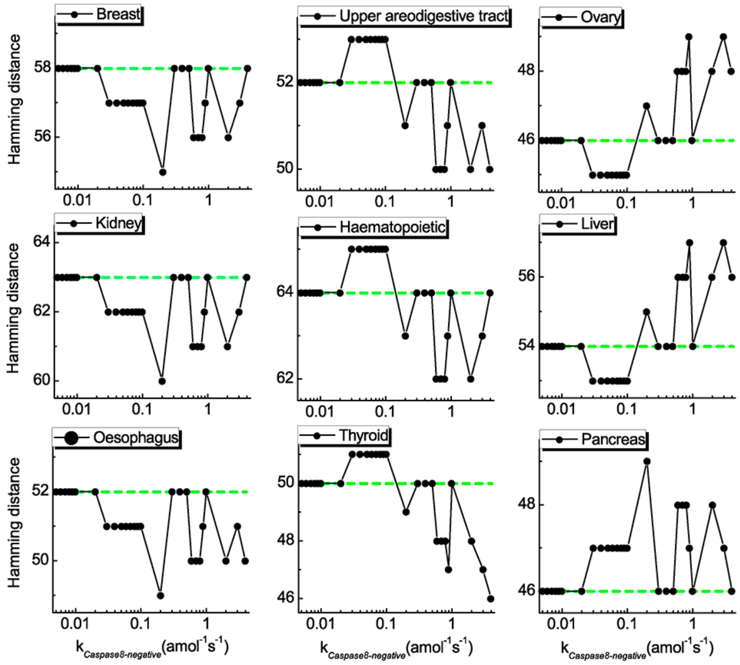

Supplementary Figure S5: HD as a function of coupling strength after considering negative feedback loops of Caspase8 on NFκB. The corresponding HDs for all the 9 cancers are calculated. For the three cancers in the first column, the majority of the HDs are smaller than those of the original model in the range of 2×10^{-2} amol^{-1}s^{-1} < $k_{caspase8\text{-}negative}$ < 4 amol^{-1}s^{-1}. For the three cancers in the second column, smaller HDs are observed in a relatively strong strength range of 2×10^{-1}amol^{-1}s^{-1}< $k_{caspase8\text{-}negative}$ < 4 amol^{-1}s^{-1}. For the two cancers (ovary and liver) in the last column, smaller HDs are observed in a relatively weak strength range of 2×10^{-2} amol^{-1}s^{-1} < $k_{caspase8\text{-}negative}$ < 10^{-1} amol^{-1}s^{-1}. However, the HD could not become smaller in the discussed range for pancreas cancer (the last subfigure). Our simulations also indicate that the HD will not be changed for all the 9 cancers after considering the positive feedback loops of Caspase8 on NFκB.

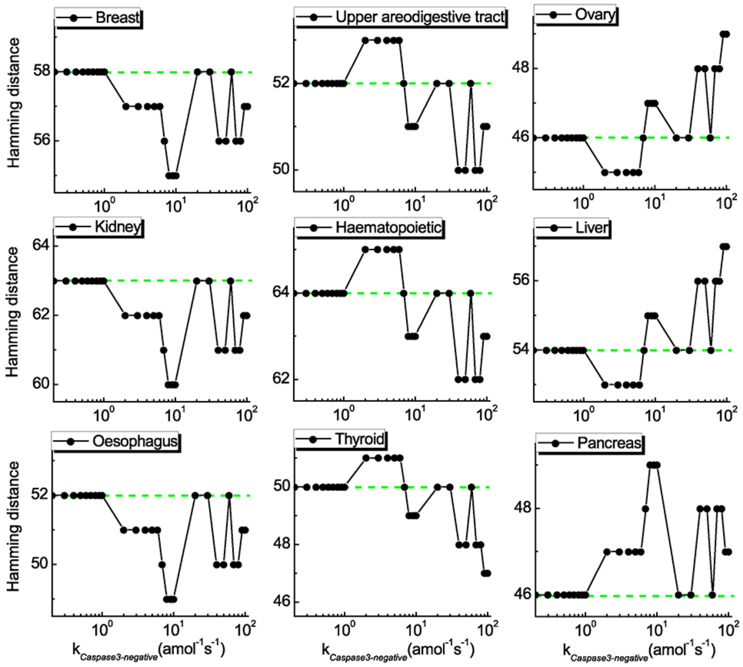

Supplementary Figure S6: HD as a function of coupling strength after considering negative feedback loops of Caspase3 on NFκB. The corresponding HDs for all the 9 cancers are calculated. The changes of HD against the negative coupling strength of Caspase3 on NFκB are similar to the case for Caspase8 on NFκB shown in Figure S5. Similarly, the HD will not be changed after considering the positive feedback loops of Caspase8 on NFκB for all the 9 cancers.

Supplementary Table S1: Parameter sensitivity spectrum and the collected cancer-related point mutation spectra. The kinetic parameters of the model are listed and described in detail. 15 of the all the 106 parameters are excluded which correspond to the proteins production rate. Parameter sensitivity spectrum and the corresponding data with the three point mutations for 9 different cancers are listed by using Boolean variable. Corresponding Hamming distances between the parameter sensitivity and corresponding point mutations are calculated in the last row for all the three point mutations of 9 different cancers. Detailed description of the model can be obtained from the Biomodels database as MODEL1112210000.

See Supplementary File 1
